# Two-year outcomes following modified transsylvian peri-insular hemispherotomy

**DOI:** 10.1007/s00381-025-06825-1

**Published:** 2025-04-25

**Authors:** Samuel B. Tomlinson, Kathleen Galligan, Sudha K. Kessler, Benjamin C. Kennedy

**Affiliations:** 1 Department of Neurosurgery, Perelman School of Medicine, University of Pennsylvania, 3400 Civic Center Blvd, Philadelphia, PA 19104 USA; 2 Division of Neurosurgery, Children’s Hospital of Philadelphia, Philadelphia, PA USA; 3 Division of Neurology, Children’s Hospital of Philadelphia, Philadelphia, PA USA; 4 Departments of Neurology and Pediatrics, Perelman School of Medicine, University of Pennsylvania, Philadelphia, PA USA

**Keywords:** Hemispherotomy, Drug-resistant epilepsy, Seizure freedom, Epilepsy surgery, Surgical technique, Lateral hemispherotomy

## Abstract

**Purpose:**

Hemispherotomy is an effective treatment for well-selected patients with drug-resistant hemispheric epilepsy. Successful hemispherotomy leading to seizure cessation has been associated with improved neurodevelopmental outcomes and reduced healthcare utilization. This study reports seizure outcomes and complications in a large, consecutive, single-surgeon series of pediatric hemispherotomy cases using a surgical approach with modifications to previously-reported techniques.

**Methods:**

All patients undergoing transsylvian peri-insular hemispherotomy for drug-resistant hemispheric epilepsy between May 2017 and April 2021 by a single surgeon were prospectively enrolled in an epilepsy surgery registry. With retrospective review of medical records, data were collected on baseline characteristics (demographics, epilepsy history, anti-seizure medications, neurodevelopmental status, EEG features, and imaging characteristics), operative complications, hospital course, and seizure outcomes (Engel scale at 12- and 24-month follow-up).

**Results:**

All 32 consecutive patients (aged 15 months–19 years) were seizure-free (Engel Class 1) 12 and 24 months after hemispherotomy. At 12 months, 31 patients (97%) had Engel Class 1A outcomes, and 1 patient (3%) had an Engel Class 1D outcome. These results were maintained through 24-month follow-up. Among 31 patients taking anti-seizure medications before surgery, 22 (71%) were weaned off all agents by 24 months. One patient (3%) developed post-operative hydrocephalus requiring ventriculoperitoneal shunt placement.

**Conclusion:**

In an etiologically heterogeneous cohort of patients undergoing hemispherotomy for drug-resistant epilepsy, a modified transsylvian peri-insular technique led to high rates of sustained seizure freedom with minimal complications.

**Supplementary Information:**

The online version contains supplementary material available at 10.1007/s00381-025-06825-1.

## Introduction

Hemispherotomy is a group of techniques for hemispheric disconnection surgery and is an effective treatment for patients with drug-resistant epilepsy caused by a variety of hemispheric pathologies [1]. Etiologies that have traditionally been considered favorable for hemispherotomy include unilateral developmental malformations (e.g., polymicrogyria, hemimegalencephaly), perinatal middle cerebral artery (MCA) stroke, Sturge-Weber syndrome, and Rasmussen’s encephalitis [2]. Successful hemispherotomy leading to seizure cessation, particularly in early life, has been associated with improved neurodevelopmental outcomes and reduced long-term healthcare utilization [3–5].

Tissue-sparing hemispherotomy has largely supplanted traditional anatomic hemispherectomy because the techniques have similar efficacy with a better safety profile, especially regarding superficial cerebral hemosiderosis and hydrocephalus [6, 7]. Lateral hemispherotomies, including variations described by Villemure and Mascott [8], Shimizu and Maehara [9], and Schramm [10], employ a lateral peri-insular approach with varying degrees of opercular and insular resection, whereas vertical hemispherotomies utilize a parasagittal corridor [11]. Despite the overall favorable efficacy of these techniques, seizure outcomes following lateral and vertical hemispherotomy remain suboptimal, with most studies documenting rates of seizure recurrence approaching or exceeding 20–30% [12–14]. Variables associated with post-operative seizure recurrence include early age of seizure onset, developmental substrate, generalized seizures, and contralateral structural abnormalities [15–17]. Completeness of disconnection, including resection of insular tissue, has also been highlighted as a key predictor of seizure freedom [24].

The purpose of this study was to describe our institutional experience performing transsylvian, peri-insular hemispherotomy in an etiologically diverse, consecutive, single-surgeon cohort. We examined 12- and 24-month seizure outcomes, complication rates, and technical insights gained through accumulated experience with this operation.

## Methods

### Study design

Patients and families were consented and enrolled prospectively in an IRB-approved, single-center observational cohort study of consecutive children and adolescents undergoing hemispherotomy for drug-resistant epilepsy from the start of the senior author’s career in May 2017 through April 2021. The a priori research plan was to identify clinical factors associated with surgical complications or seizure recurrence.

### Presurgical evaluation

All patients underwent presurgical evaluation including clinical assessment by a pediatric epileptologist, video electroencephalogram (EEG), and contrast-enhanced magnetic resonance imaging (MRI). Selected patients also underwent preoperative magnetoencephalography (MEG), neuropsychological assessment, positron emission tomography (PET), functional MRI (fMRI), and stereoelectroencephalography (sEEG). Case review at a multidisciplinary epilepsy surgery conference preceded a recommendation for hemispherotomy.

### Surgical technique

**Fig. 1:**
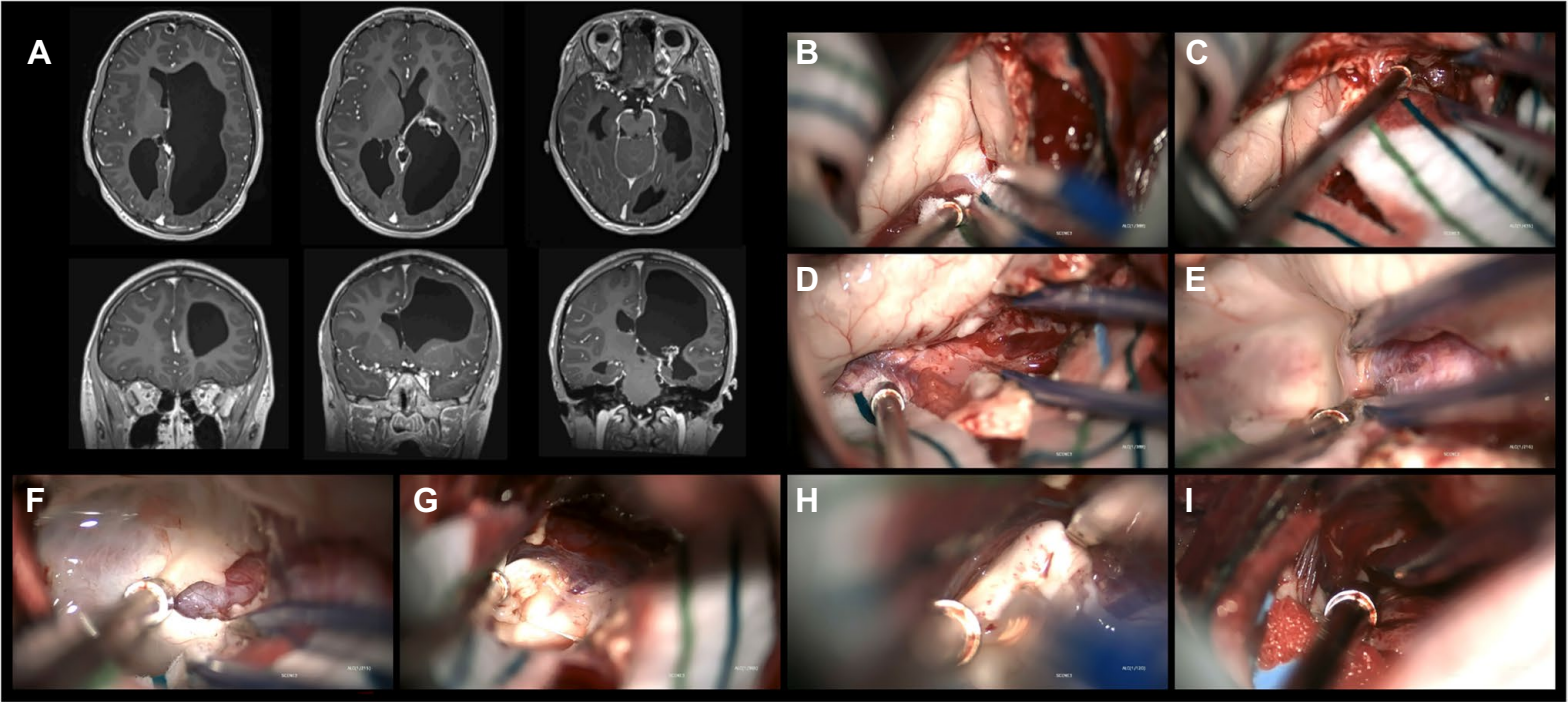
Illustrative case demonstrating transsylvian peri-insular hemispherotomy. **A** Pre-operative T1-weighted post-contrast axial and coronal MRIs demonstrating left-sided porencephaly, ventricular dilation, and callosal thinning. **B** Identification of choroidal point. **C** Amygdala disconnection: proximal MCA viewed through the temporal pia into the Sylvian fissure. **D** Medial pia contiguous with opened choroid fissure, completing the amygdala disconnection. **E** Lifting of the fornix off the choroid fissure, which is followed along the medial pia into the splenium. **F** Transection of fornix along the medial pia. **G** Continuation of forniceal transection to the white-matter-pia interface of the ipsilateral splenium. **H** Ipsilateral transection of callosal body. **I** Fronto-basal disconnection: visualization of posterior pia of orbitofrontal gyrus marked by lenticulostriate arteries exiting proximal MCA to enter basal ganglia

**Fig. 2:**
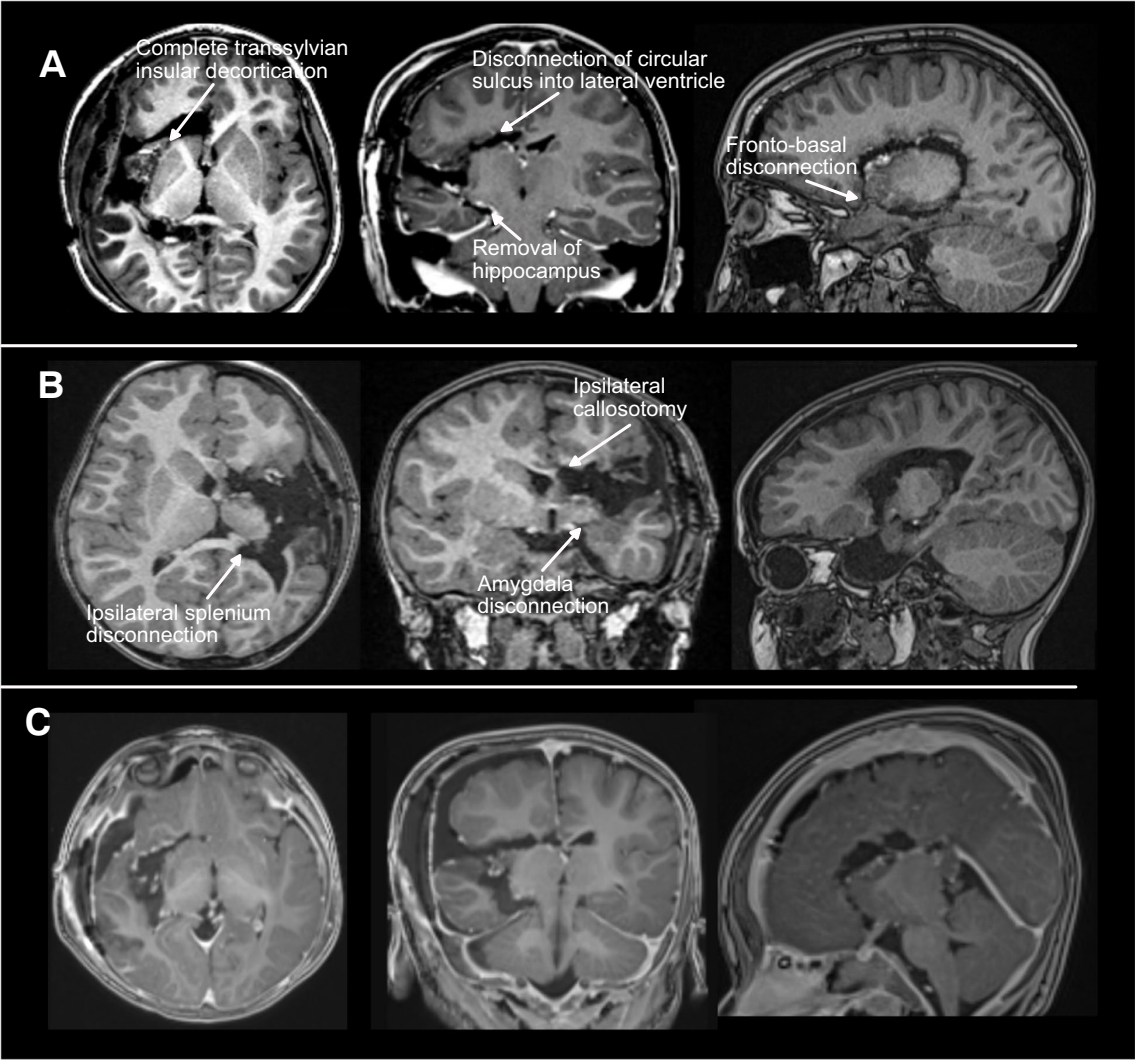
Representative post-operative MRIs following modified transsylvian hemispherotomy. T1-weighted axial, coronal, and sagittal images are displayed. **A** 11-year-old with Rasmussen’s encephalitis. **B** 4-year-old with perinatal L MCA stroke. **C** 15-month-old with hemispheric dysplasia. Radiographic correlates of several key disconnection steps are annotated

All patients underwent transsylvian peri-insular hemispherotomy (PIH) by the senior author, with additional modifications when anatomically necessary (Fig. 1). This technique is most similar to the procedure described by Schramm [10]. The operation begins with a linear incision and an approximately 3–4-cm circular craniotomy centered over the Sylvian fissure, followed by a C-shaped dural opening. Using a microscope, the Sylvian fissure is widely split to expose the entire insula and circular sulcus via the transsylvian corridor. The circular sulcus is traversed to enter the temporal horn of the lateral ventricle. The amygdala is disconnected from the globus pallidus using subpial dissection along the posterior aspect of the proximal MCA to the superior pia of the amygdala, continuing to the choroidal point. At this time, pathological specimens are sent, with or without an *en bloc* resection of the hippocampal head and body. Dissection continues along the circular sulcus permitting entry into the atrium. Regardless of whether the hippocampal head and body have been resected, the hippocampal tail and fornix are transected along the medial pia up to the splenium where the white matter-pia interface in a mediolateral orientation can be appreciated. Then, segmentally, approximately 2 cm at a time, the circular sulcus is traversed into the body, then frontal horn of the lateral ventricle. Care is taken to spare most M3 branches and work around them. At each segment, the white matter-pia interface is followed rostrally, continuing the callosotomy. This white matter-pia interface is maintained on the ipsilateral hemisphere on the ventral side of the callosal sulcus, avoiding midline vessels and exposure or potential injury of the contralateral hemisphere (Fig. 2b). Fronto-basal disconnection is performed from the tip of the frontal horn to the posterior pia of the orbitofrontal gyrus as the posterior boundary inferiorly, and the anterior circular sulcus laterally. Subpial resection of the insula is also performed. Throughout surgery, care is taken to promptly irrigate and stop bleeding, as well as pack off areas in which disconnection has been completed with thrombin-soaked cotton balls. An external ventricular drain (EVD) is tunneled supero-posterior to the incision, secured at the skin edge, and left subdurally. Post-operatively, the patient is brought to the pediatric intensive care unit for recovery. A non-contrast brain MRI obtained on the first post-operative day is reviewed to confirm complete disconnection. The EVD is drained at a low pressure and removed on post-operative day 3.

### Clinical data extraction and analysis

Preoperative data were collected from electronic medical records in retrospective fashion. Variables of interest included patient demographics, epilepsy history, anti-seizure medications (ASM), functional status, neurodevelopmental status, EEG features, and imaging characteristics. Both EEG and imaging review included attention to the presence of contralateral abnormalities. Details regarding operative technique, intraoperative findings, and immediate post-operative events were obtained from the surgical case log. Histopathologic details were ascertained from pathology reports. Post-operative evaluation included multiple clinic visits after surgery with both the neurology and neurosurgery teams. Formal seizure outcome classification was made by the patient’s primary neurologist without influence from this study. Patients and families were thoroughly interviewed to elucidate possible auras or seizures of any semiology. Seizure outcomes were formally graded at 12- and 24-months according to the modified Engel classification scale [18]. Change in ASM burden before and after hemispherotomy was quantitatively assessed at the cohort level using the Wilcoxon signed-rank test (*α* = 0.05).

Completeness of anatomic disconnection was assessed via review of volumetric T1-weighted post-operative MRI in all three planes. Specifically, we assessed for complete disconnection of the amygdala from the Sylvian fissure to choroidal point, hippocampal tail to the medial pia into the splenium, complete transection of the corpus callosum, disconnection through the circular sulcus into the lateral ventricle, and fronto-basal disconnection from the posterior pia of the orbitofrontal gyrus and gyrus rectus to the anterior insula. Finally, completeness of resection of the insular cortex was evaluated (Fig. 2).

## Results

### Baseline characteristics

Clinical characteristics of the cohort are presented in Table 1. Thirty-two patients underwent hemispherotomy during the study window (59.4% male). The median age at seizure onset was 2.5 years (range: 3 days to 11 years). The median age at time of surgery was 8.0 years (range, 15 months to 19 years). Etiology of epilepsy was perinatal stroke in 28% (*n* = 9), hemispheric dysplasia/malformation in 25% (*n* = 8), Rasmussen’s encephalitis in 22% (*n* = 7), Sturge-Weber syndrome in 13% (*n* = 4), traumatic brain injury in 9% (*n* = 3), and postnatal stroke in 3% (*n* = 1). The left hemisphere was disconnected in 53% of patients (*n* = 17).

**Table 1:** Clinical attributes of study population (*N* = 32)

Study population (*N* = 32)	*N* (%)
**Sex**	
Male	19 (59.4)
Female	13 (40.6)
**Hemispherotomy side**	
Left	17 (53.1)
Right	15 (46.9)
**Etiology**	
Perinatal stroke	9 (28.1)
Hemispheric dysplasia	8 (25.0)
Rasmusssen's encephalitis	7 (21.9)
Sturge-Weber syndrome	4 (12.5)
Traumatic brain injury	3 (9.4)
Post-natal stroke	1 (3.1)
**Seizure semiology**	
Focal	25 (78.1)
Focal to bilateral tonic–clonic	15 (46.9)
Infantile spasms	6 (18.8)
**Electrographic onset**	
Ipsilateral/broad (non-localizing)	20 (62.5)
Ipsilateral/focal (regional)	5 (15.6)
Independent contralateral	0 (0)
**MRI abnormalities**	
Ipsilateral-only	22 (68.8)
Bilateral	10 (31.3)
**12-month outcome (*N* = 32)**	
Engel I (A–D)	32 (100)
Engel II or higher	0 (0)
**24-month outcome (*N* = 31)**	
Engel I (A–D)	31 (100)
Engel II or higher	0 (0)

Abbreviations: magnetic resonance imaging (MRI)

Preoperative neurologic examination identified contralateral hemiparesis in 94% (*n* = 30), involving the upper extremity in 84% (*n* = 27), lower extremity in 72% (*n* = 23), and the face in 31% (*n* = 10). Developmental status at the time of surgery was assessed as normal/age-appropriate in 19% (*n* = 6). Most patients (26/32, 81.3%) were ambulatory at the time of surgery. Patients who were not ambulatory preoperatively were between ages 15 months and 4 years at time of surgery.

Pertinent electrographic and radiologic findings are summarized in Table 2. Sixty-six percent of patients (*n* = 21) had more than one typical seizure semiology at the time of hemispherotomy. Independent interictal epileptiform discharges in the non-operated hemisphere were observed in 19% (*n* = 6). Most patients (63%, *n* = 20) exhibited lateralized but broad, non-localizing electrographic ictal onset. No patients in the cohort exhibited independent contralateral electrographic seizure onset. Radiographic structural abnormalities were identified in the contralateral hemisphere in 31% (*n* = 10). Contralateral findings were heterogeneous across patients, including cortical malformations, encephalomalacia, atrophy, diffusion restriction, and other non-specific signal changes.

**Table 2:** Individual patient characteristics

ID	Sex	Hand dominance	Age (mo) at seizure onset	Age at surgery	Etiology	Surgical laterality	EEG	MRI	Contralateral abnormalities	Prior surgical procedure for epilepsy
1	M	R	100	9 yrs	TBI	R	Vertex epileptiform discharges; R hemispheric attenuation	Hemispheric cystic encephalomalacia/gliosis	EEG: none MRI: none	No (craniotomy for TBI)
2	M	R	24	10 yrs	TBI	R	Background asymmetry; focal intermittent R posterior slowing; R temporal focal sharps	Hemispheric cystic encephalomalacia/gliosis	EEG: none MRI: none	No (craniotomy for TBI)
3	M	L	3	8 yrs	TBI	L	L hemispheric attenuation; rare R frontal epileptiform discharges	Hemispheric cystic encephalomalacia/gliosis	EEG: rare R frontal epileptiform discharges MRI: none	No (craniotomy for TBI)
4	F	R	96	9 yrs	RE	L	L frontal ictal onset; L hemispheric background disorganization; frequent L frontocentral sharps	Patchy/confluent L hemispheric T2 signal prolongation, volume loss, ex vacuo ventriculomegaly	EEG: slowing MRI: none	Yes (seizure focus resection with electrocorticography)
5	M	R	8	8 yrs	Hemispheric dysplasia (hemimegalencephaly)	R	Continuous R frontal/hemispheric epileptiform discharges; generalized paroxysmal fast activity in sleep (ispilateral predominant)	R hemimegalencephaly, dysmorphic gyration/polymicrogyria; R periventricular gray matter heterotopia	EEG: none MRI: small area of polymicrogyria L parasagittal parieto-occipital lobe; L MTS; subcentimeter T2 abnormality within the posteromedial L thalamus suspicious for low-grade glioma	No
6	F	R	4	21 mo	Hemispheric dysplasia; Aicardi syndrome; polymicrogyria	R	Hypsarrhythmia (ipsilateral predominant)	R periventricular cystic structures; agenesis of corpus callosum with polymicrogyria	EEG: hypsarrhythmia (ipsilateral predominant) MRI: none	No
7	F	L	0.1	18 mo	Perinatal stroke (L MCA)	L	Hypsarrhythmia (ipsilateral predominant)	Hemispheric cystic encephalomalacia/gliosis, ex vacuo ventriculomegaly	EEG: hypsarrhythmia (ipsilateral predominant) MRI: nonspecific T2 signal along the R lateral and fourth ventricles	No
8	M	L	36	11 yrs	Perinatal stroke, IVH prematurity	L	Occasional L temporal/central sharps	Extensive periventricular leukomalacia; L MTS	EEG: slowing MRI: R posterior fossa/periventricular cyst	No (extensive prior shunt/fenestration history for hydrocephalus)
9	M	L	12	14 mo	Perinatal stroke (L MCA)	L	L hemi-hypsarrhythmia; L frontal ictal onset	Hemispheric cystic encephalomalacia/gliosis, ex vacuo ventriculomegaly	EEG: none MRI: none	No
10	M	Mixed	52	4 yrs	RE	R	R hemispheric slowing; frequent frontocentral sharps	Post-craniotomy changes with resection of R anterior temporal lobe	EEG: none MRI: none	Yes (R anterior temporal lobe, insula, and orbitofrontal gyrus resection)
11	F	R	30	3 yrs	Hemispheric dysplasia	R	R hemispheric slowing; R paroxysmal fast activity; R hemispheric ictal-interictal continuum	Diffuse R hemispheric cortical dysplasia	EEG: none MRI: none	Yes (R occipital lobectomy)
12	M	L	125	14 yrs	SWS	R	Frequent R frontotemporal sharps with secondary generalization; R frontotemporal ictal onset	Prior R temporal lobectomy; gliosis in residual R hippocampus; R hemispheric volume loss	EEG: none MRI: none	Yes (R temporal lobectomy)
13	M	R	6	14 yrs	Postnatal stroke (R MCA)	R	R centro-temporo-parietal attenuation; R posterior temporal sharps	R MCA distribution cystic encephalomalacia/gliosis, ex vacuo ventriculomegaly	EEG: L temporal sharps; diffuse L hemispheric fast activity; intermittent L temporal slowing MRI: none	No
14	F	L	34	5 yrs	Perinatal stroke (L MCA)	L	L hemispheric slowing with abundant sharps (predominately frontal)	Hemispheric cystic encephalomalacia/gliosis, ex vacuo ventriculomegaly	EEG: none MRI: nonspecific T2 periventricular signal	No
15	F	L	137	11 yrs	Perinatal stroke (L MCA)	L	Not available	Hemispheric cystic encephalomalacia/gliosis, ex vacuo ventriculomegaly	EEG: none MRI: none	No
16	M	L	1.3	15 mo	Hemispheric dysplasia	L	L hemispheric slowing with abundant sharps	Extensive L hemispheric dysplasia, partial callosal agenesis, R lateral ventriculomegaly	EEG: slowing MRI: none	No
17	F	L	48	4 yrs	RE	L	Abundant multifocal L hemispheric sharps; ictal onset (SEEG): L superior parietal lobule, hippocampus/amygdala, leg motor, hand motor	L frontal subcortical T2 prolongation; L MTS	EEG: none MRI: none	No
18	F	R	61	11 yrs	RE	R	Occasional R fronto-centro-parietal sharps; R frontal ictal onset	Diffuse R hemispheric atrophy/volume loss and white matter T2 signal	EEG: occasional L frontal sharps MRI: none	No
19	F	R	46	4 yrs	Perinatal stroke, IVH prematurity	R	Diffuse R hemispheric sharps; ictal onset (SEEG): hippocampal/temporo-occipital	Hemispheric cystic encephalomalacia/gliosis, ex vacuo ventriculomegaly	EEG: none MRI: nonspecific subcortical L superior frontal T2 prolongation	No
20	F	Unknown	1.5	15 mo	Hemispheric dysplasia	R	Continuous R hemispheric epileptiform discharges, ictal-interictal continuum	Extensive diffuse T2 hyperintense signal throughout R hemisphere; bilateral T2 prolongation within thalami and hippocampi; R parietal gyral disorganization	EEG: none MRI: nonspecific hippocampal/thalamic and medial occipital T2 prolongation and diffusion restriction	No
21	M	L	12	2 yrs	SWS	L	Continuous L posterior quadrant focal slowing; rare L occipital/temporal epileptiform discharges; L temporal and occipital ictal onset	Posterior L hemispheric atrophy, calcifications, leptomeningeal enhancement, and mild diffusion restriction; enhancement of L lateral ventricle choroid plexus	EEG: none MRI: none	No
22	M	R	12	2 yrs	Hemispheric dysplasia	R	Frequent R frontotemporal epileptiform discharges; epileptic spasms with R posterior quadrant onset	Extensive cortical dysplasia within the R superior, middle and inferior frontal gyri, R insula	EEG: occasional L frontotemporal sharps MRI: none	No
23	F	Mixed	54	11 yrs	Hemispheric dysplasia	L	L posterior quadrant focal slowing; L central/frontotemporal spikes; L central ictal onset	L occipito-temporal encephalomalacia and gliosis, ex vacuo ventriculomegaly	EEG: none MRI: none	Yes (L temporal occipital lobectomy)
24	M	L	7	15 mo	Perinatal stroke (L MCA)	L	L hemispheric slowing and background disorganization; focal L temporooccipital, frontopolar, and central vertex spikes	Hemispheric cystic encephalomalacia/gliosis, ex vacuo ventriculomegaly	EEG: none MRI: chronic focal encephalomalacia/gliosis in the cortical/subcortical R parietal lobe consistent with chronic infarct; generalized volume loss R posterior cerebral hemisphere, R periventricular leukomalacia	No
25	F	R	128	11 yrs	RE	R	R hemispheric slowing; R central/parietal focal epileptiform discharges; R central ictal onset	Bifrontal nonenhancing T2/FLAIR hyperintensity. Gyral expansion and signal alteration within R mesial temporal lobe, operculum and insula	EEG: none MRI: bifrontal T2/FLAIR signal change	No
26	M	R	45	9 yrs	Perinatal stroke (R MCA)	R	Near-continuous R > L frontal slowing; abundant central vertex/R frontotemporal sharps; R frontal/central ictal onset	R MCA distribution cystic encephalomalacia/gliosis, ex vacuo ventriculomegaly	EEG: frontal slowing, ipsilateral predominant MRI: L periatrial gliosis and focal atrophy	No
27	M	R	48	8 yrs	RE	L	L hemispheric slowing; continuous focal motor seizure activity associated with L hemispheric rhythmic slowing; epilepsia partialis continua	L hemispheric scattered patchy regions of signal abnormality and volume loss, ex vacuo ventriculomegaly	EEG: frontotemporal slowing MRI: none	No
28	M	R	42	3 yrs	RE	L	Interictal discharges (SEEG) from insula with frontal propagation; additional independent spike populations arising from hippocampus and amygdala; temporal/insular ictal onsets	Extensive confluent L temporal/amygdala/posterior insular T2 signal hyperintensity	EEG: none MRI: none	No
29	F	L	130	19 yrs	Hemispheric dysplasia	L	Abundant L temporal > central sharps; L predominant generalized polyspike-and-wave discharges; diffuse (nonlateralizing) ictal onset	Diffuse L hemispheric atrophy/volume loss; L periventricular nodular heterotopia; polymicrogyria, schizencephaly	EEG: rare R frontopolar sharps; diffuse ictal onset MRI: none	No
30	M	L	27	14 yrs	SWS	L	Prolonged L temporal ictal activity; diffuse generalized slowing	Extensive gyriform calcifications and enhancement involving L temporoparietal and occipital regions; L hemispheric atrophy/volume loss	EEG: diffuse slowing MRI: none	No
31	M	L	55	15 yrs	Perinatal stroke (L MCA)	L	L hemispheric slowing	Hemispheric cystic encephalomalacia/gliosis	EEG: none MRI: none	No
32	M	R	10	20 mo	SWS	R	R hemispheric slowing; R posterior quadrant focal status epilepticus	R hemisphere atrophy in association with leptomeningeal enhancement/pial angiomatosis	EEG: rare L temporal/central focal slowing MRI: Leptomeningeal enhancement involving the L superior frontal gyrus and parasagittal midline	No

Abbreviations: *M *Male, *F* Female, *MO* Months, *yrs* Years, *L* left, *R* Right, *TBI* Traumatic brain injury, *MTS* Mesial Temporal Sclerosis, *MCA* middle cerebral artery, *IVH* Intraventricular hemorrhage, *RE* Rasmussen's encephalitis, *SWS* Sturge-Weber syndrome, *SEEG* Stereoelectroencephalography

### Seizure outcomes

At 12 months after hemispherotomy, all patients met criteria for Engel Class 1 seizure freedom (Table 3). One patient (Engel 1A at 12 months) relocated prior to the 24-month visit and was lost to follow-up. As a result, at 24-month follow-up, 31/31 patients met Engel Class 1 criteria. One patient had an Engel Class 1D outcome due to breakthrough seizures during a rapid taper of one of eight ASM. This patient had drug-resistant epilepsy due to a hemispheric malformation and presented to our institution for surgical intervention after a month-long period of status epilepticus. Imaging showed extensive diffusion restriction injury throughout the ipsilateral hemisphere in addition to the contralateral hippocampus and some areas of contralateral neocortex. After this single episode of seizure recurrence, additional ASM were successfully weaned down to two with no further seizure recurrences by 24 months.

**Table 3:** Individual patient outcomes at 12- and 24-months after surgery

ID	Engel 12 mo	Engel 24 mo	ASM pre-op (#)	ASM 24 mo (#)	Post-surgical complications
1	1 A	1 A	1	0	Post-operative pancreatitis and associated pleural effusion requiring chest tube
2	1 A	1 A	1	0	N/A
3	1 A	1 A	1	0	N/A
4	1 A	1 A	1	2	N/A
5	1 A	1 A	4	1	N/A
6	1 A	1 A	0	0	N/A
7	1 A	1 A	1	0	N/A
8	1 A	1 A	3	0	Extensive pre-hemispherotomy history of shunt revisions and endoscopic and open fenestrations; required multiple shunt and fenestration revisions during follow-up period
9	1 A	1 A	2	0	Post-operative hydrocephalus requiring ventriculoperitoneal shunt placement
10	1 A	1 A	4	1	N/A
11	1 A	Lost to follow-up	3	0 (12 mo)	N/A
12	1 A	1 A	5	0	N/A
13	1 A	1 A	2	0	Post-operative pancreatitis managed medically
14	1 A	1 A	2	0	N/A
15	1 A	1 A	1	0	N/A
16	1 A	1 A	3	2	N/A
17	1 A	1 A	3	0	N/A
18	1 A	1 A	3	0	N/A
19	1 A	1 A	2	0	N/A
20	1D	1D	8	2	N/A
21	1 A	1 A	1	0	N/A
22	1 A	1 A	1	0	N/A
23	1 A	1 A	2	1	N/A
24	1 A	1 A	1	0	N/A
25	1 A	1 A	4	1	N/A
26	1 A	1 A	2	0	N/A
27	1 A	1 A	2	0	N/A
28	1 A	1 A	5	0	N/A
29	1 A	1 A	4	1	N/A
30	1 A	1 A	6	0	N/A
31	1 A	1 A	2	1	N/A
32	1 A	1 A	2	0	N/A

### Anti-seizure medications

**Fig. 3:**
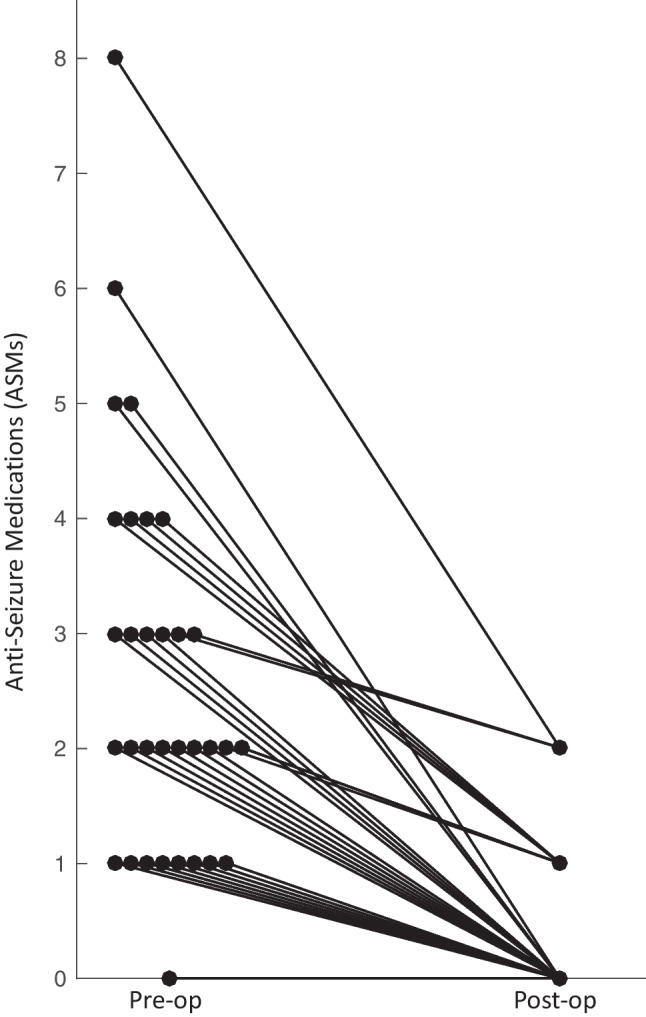
Peri-operative change in anti-seizure medications (ASM). Twenty-two of 31 patients (71%) taking ASM at the time of hemispherotomy had discontinued all ASM by 24-month follow-up

Prior to surgery, patients were prescribed a median of 2 ASMs (range 0–8, interquartile range 1–3.5). Among the 31 patients taking ASMs prior to surgery, all 31 (100%) exhibited a reduction in ASM number post-operatively (Table 3). The median number of ASMs declined over 2 years to a median of 0 (range, 0–2; Wilcoxon signed-rank test, *p* < 0.0001) (Fig. 3). At 24-months follow-up, 22/31 patients (71%) were off all ASMs.

### Surgical outcomes

MRI demonstrated complete hemispheric disconnection in every case. The median number of days from surgery to medical clearance for discharge was 10 (range, 5–36). Ten patients (31.3%) were discharged to home from the hospital (age range, 1–14 years; median, 5 years), while the remaining 22 patients were discharged to inpatient rehabilitation.

### Postsurgical complications

One patient (1/32, 3.1%) developed new-onset hydrocephalus requiring ventriculoperitoneal (VP) shunt placement after hemispherotomy. One patient with a history of intraventricular hemorrhage (IVH) of prematurity and complex, loculated hydrocephalus leading to multiple shunt revisions and fenestrations prior to hemispherotomy had several subsequent shunt revisions and fenestrations in the follow-up period. No other patient experienced re-operation, infection, wound issue, contralateral injury, or other surgical complication throughout the follow-up period. Two patients developed transient post-operative pancreatitis that abated with medical management. No patient developed any endocrinopathy, including diabetes insipidus or other hypothalamic or pituitary dysfunction. All patients who were ambulatory pre-operatively retained or regained ambulation after inpatient rehabilitation.

## Discussion

In this study, we reviewed our institutional experience performing transsylvian, peri-insular hemispherotomy in a consecutive series of 32 children with heterogeneous seizure substrates. Hemispherotomy is not a new or uncommon procedure for epilepsy with hemispheric pathology because of its high degree of efficacy [13], but there are still unanswered questions about how to optimize seizure outcomes and minimize complications. Because the procedure often introduces deficits or worsens a patient’s existing functional deficits (e.g., hemiparesis, hemianopsia, and language impairment), it is undertaken only when the likelihood of seizure freedom, and subsequent protection of the intact hemisphere, clearly outweighs the impact of deficits over the long term. The efficacy of hemispherotomy for drug-resistant seizures exceeds that of other surgical resection types overall [2], but across centers and series, seizure freedom rates are generally below 80% (see Table S1 for review of prior case series). In a 32-center study which included 1267 cases between 1986 and 2018 to develop a Hemispheric Surgery Outcome Prediction Scale (HOPS), 74% of patients had Engel Class 1 outcomes at 24 months [15]. Other meta-analyses evaluating 1528 cases from 56 studies [16] and 1161 patients from 29 studies [17] reported seizure freedom rates of 73% and 73.4%, respectively, at last clinical follow-up.

Seizure relapse after hemispherotomy is a complex phenomenon with many potential contributors. Regarding preoperative characteristics, the factor most consistently associated with seizure outcome in the literature is the etiology of hemispheric pathology: patients with acquired early-life injury (e.g., perinatal MCA ischemic stroke) have the highest long-term seizure freedom rates, whereas patients with malformations of cortical development (e.g., hemimegalencephaly) have the lowest [17]. Institutional differences in post-operative clinical care, especially with regard to ASM management, could also contribute to variations in seizure outcomes. Our series represents the typical distribution of seizure substrates encountered at a large epilepsy referral center, and our inclusion of all consecutive patients throughout the study window makes it unlikely that patient selection alone accounts for the high seizure freedom rate observed in our study. Though we cannot directly compare post-hemispherotomy medication management in our series to others, our cohort had a high degree of ASM discontinuation prior to the 24-month endpoint, which suggests that our results are not confounded by cases of likely eventual seizure recurrence masked by ASM therapy.

**Fig. 4:**
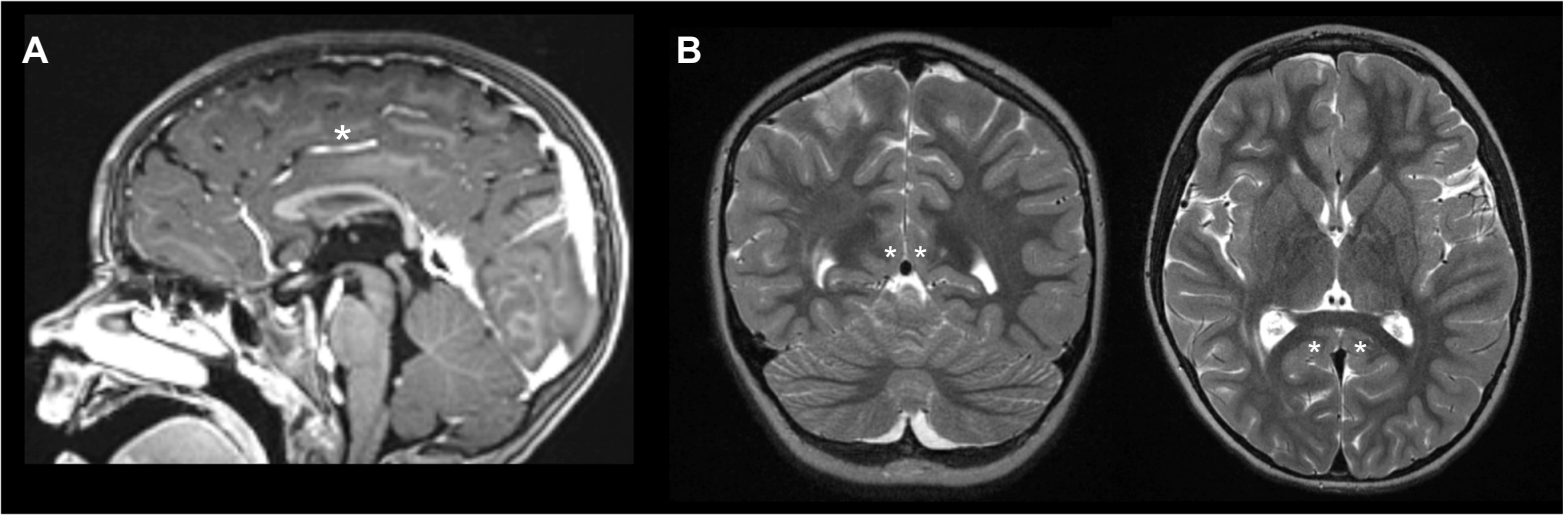
Examples of misleading extrinsic landmarks for disconnection. **A** In this example, the major continuation of the anterior cerebral artery lies within the cingulate sulcus as a callosomarginal artery (asterisk) and does not lead to the corpus callosum. Following this artery may result in failure to disconnect a pathologic cingulate gyrus from the contralateral side. **B** The falcotentorial junction in this example does not lead to the splenium of the corpus callosum, which may result in failure to disconnect the posterior cingulate (asterisks)

Various surgical factors have been examined in relation to seizure outcomes following hemispherotomy, although it should be noted that no surgical technique can safeguard against the emergence of contralateral seizure foci resulting in seizure relapse. Regarding surgical technique, one factor which has been noted previously as a key predictor of long-term seizure freedom is completeness of disconnection [19, 20]. One large meta-analysis found that the rate of revision surgery for incomplete disconnection and/or seizure recurrence following hemispherotomy was 19/480 (4.0%) [21]. Another study examining unsuccessful hemispherotomy necessitating revision identified residual connected substrate in 5/6 patients requiring reoperation [22]. In our experience, the lateral transsylvian hemispherotomy pioneered by Schramm and colleagues [10] affords an excellent corridor through which to ensure complete disconnection. Techniques within this lineage utilize a lateral peri-insular window to access the lateral ventricle, corpus callosum, mesial temporal lobe, and frontobasal structures. Our approach to this technique places emphasis on intrinsic brain landmarks (e.g., white matter-pia interface of the corpus callosum, posterior pia of the orbitofrontal gyrus, superior pia of the amygdala) rather than dural or vascular landmarks (e.g., tentorium, falx, pericallosal artery, and proximal ACA). Utilizing intrinsic brain landmarks as described avoids potential pitfalls of the inherent anatomical variability among patients in terms of the relationship between brain tissue and support structures. For example, in patients without a robust pericallosal artery, if one were to follow the major artery to complete the callosotomy, and it lies in the cingulate sulcus as a callosomarginal artery, a potentially pathologic ipsilateral cingulate gyrus may remain connected to the contralateral side (Fig. 4a). Similarly, a falcotentorial junction can be followed more posteriorly and superiorly, i.e., more eccentrically, than the posterior callosum, which could result in either the same type of incomplete disconnection of the posterior cingulate, or a remainder of a hippocampal tail still connected to the fornix, mammillary body, and hippocampal commissure (Fig. 4b). These intrinsic anatomic landmarks are present in all patients, even those with hemimegalencephaly and other structural abnormalities. Although these cases may be more challenging and require more preoperative study, these landmarks remain highly reliable across patients for ensuring complete disconnection. Other nuances include disconnection of the amygdala up to the superior pia of the uncus, as well as a focus not on necessarily resecting the hippocampus but on a very posterior disconnection of the hippocampal tail and fornix right to the splenium, minimizing the amount of tissue removed during the procedure. Furthermore, when the Sylvian fissure is split widely enough to appreciate the entirety of the circular sulcus, this can optimize the view of the entire insula, allowing potentially more complete resection of insular gray matter. Having the anterior circular sulcus fully exposed and using the posterior pia of the orbitofrontal gyrus and gyrus rectus as landmarks for the frontobasal disconnection may avoid leaving posterior orbitofrontal gyrus or subgenual cingulate connected to the deep structures, while also helping delineate the anterior aspect of the hypothalamus, potentially preventing hypothalamic dysfunction.

Another notable finding in our study is the low rate of post-operative hydrocephalus, which is the most frequently-reported complication following hemispheric epilepsy surgery [23], and one which may impose a substantial long-term burden of medical intervention. In a retrospective pooled analysis of 736 patients from 15 pediatric epilepsy centers undergoing hemispheric surgery followed for an average of 2.9 years, 23% of patients developed hydrocephalus requiring shunt placement [24]. Importantly, while the time interval between surgery and shunt placement in that study ranged from immediately post-operative to 8.5 years, the majority (119/162, 73.5%) occurred within 90 days of surgery. Technical and patient factors contributing to new-onset hydrocephalus after hemispherotomy remain controversial. Although our study is neither designed nor powered to answer this question, we have adopted the following technical nuances at our center in an effort to minimize postoperative hydrocephalus: (1) use of a wide Sylvian fissure split to access critical substrates for disconnection with the minimum amount of brain resection; (2) use of the smallest possible craniotomy to minimize disruption of arachnoid and arachnoid granulations; (3) meticulous hemostasis throughout the case and preservation of intact pial planes to prevent blood from entering the subarachnoid spaces of the basal and interhemispheric cisterns, as well as the ventricles; and (4) 3 days of post-operative EVD drainage to protect the wound and remove blood from the cerebrospinal fluid. It is important to note that no single unifying mechanism accounting for post-hemispherotomy hydrocephalus (PHH) has been described. Previous literature points to both theoretical and population-level risk factors for PHH, including volume of resected tissue, size of craniotomy, disruption of arachnoid granulations, and entry of blood and debris to the subarachnoid space [24]. Excessive CSF production has also been raised as a potential contributor, and at least one group has incorporated routine choroid plexus cauterization (CPC) during hemispheric epilepsy surgery [25]. The low shunt rate observed in our series, in which CPC was not performed, argues against the necessity of routine CPC to reduce CSF production as a maneuver to decrease the risk of PHH.

Strengths of our study include the size of the cohort for the number of years reported and the low loss to follow-up. All outcomes for this cohort were collected in the context of an in-person evaluation by an epileptologist at our institution. The cohort included all patients undergoing hemispherotomy in the specified time-period, from the start of the senior author’s career, prospectively enrolled, to minimize selection bias. Our total cohort follow-up time of 24 months is a relative strength compared to some studies with variable and short follow-up, but a relative weakness given prior literature indicating a gradual decline in seizure freedom rates over a longer follow-up period. For example, the HOPS study reported seizure freedom rates of 83%, 74%, and 66% at 1, 2, and 5 years, respectively [16]. Continued long-term clinical follow-up of our cohort beyond 24 months will be essential for determining whether seizure recurrence will manifest at a later stage.

## Supplementary Information

Below is the link to the electronic supplementary material.

Supplementary file1 (DOCX 34.6 KB)

## Data Availability

No datasets were generated or analysed during the current study.
