# Supplementary material for: Two-year outcomes following modified transsylvian peri-insular hemispherotomy
Source: Childs Nerv Syst. 2025 Apr 25;41(1):168. doi: 10.1007/s00381-025-06825-1 (PMC12031986; doi:10.1007/s00381-025-06825-1)
Supplement: Supplementary file 1 — Supplementary file1 (DOCX 34.6 KB) [file 381_2025_6825_MOESM1_ESM.docx]

| **Ref** | **Author** | **Institution(s) (Country)** | **Surgical Technique(s)** | **Date Range** | **N** | **Seizure Follow-up (as reported)** | **% Seizure Free (metric)** | **Hydro/shunt (if reported)** |
| --- | --- | --- | --- | --- | --- | --- | --- | --- |
| **[1]** | **Abraham (2019)** | Christian Medical College (India) | Lateral peri-insular hemispherotomy | 2005-2016 | 45 | Mean: 4 years | 93.2% (Engel I) | 2/44 (4.5%) |
| **[2]** | **Althausen (2012)** | University of Bonn Medical Center (Germany) | Transsylvian, transcortical and modified hemispherectomy | 1988-2007 | 61 | >1 year | 73.8% ('seizure free') | NR |
| **[3]** | **Arifin (2019)** | Kariadi Hospital; Telogorejo Hospital (Indonesia) | Lateral peri-insular hemispherotomy | 1999-2019 | 16 | >2 years | 62.5% (Engel I) | NR |
| **[4]** | **Basheer (2007)** | British Columbia Children’s Hospital (Canada) | Hemidecordication, lateral peri-insular hemispherotomy | 1993-2004 | 24 | >1 year | 79.2% (Engel I) | 2/24 (8.3%) |
| **[5]** | **Buckley (2014)** | Seattle Children’s Hospital (USA) | Lateral peri-insular hemispherotomy | 1997-2012 | 39 | 0.3-10.6 years | 71.8% ('seizure free') | 6/39 (15.4%) |
| **[6]** | **Caraballo (2011)** | Hospital Nacional de Pediatría Prof. Dr. Juan P. Garrahan (Argentina) | Functional hemispherectomy, peri-insular hemispherotomy | 1990-2010 | 45 | >1 year | 73.5% (Engel I) | 1/45 (2.2%) |
| **[7]** | **Cats (2007)** | University Medical Center Utrecht (Netherlands) | Lateral transsylvian hemispherotomy, peri-insular deafferentation | 1992-2004 | 28 | 0.5-11 years | 78.6% (Engel I) | 2/28 (7.1%) |
| **[8]** | **Chandra (2008)** | All India Institute of Medical Sciences (India) | Vertical parasagittal hemispherotomy, lateral peri-insular hemispherotomy | 2001-2007 | 19 | 0.6-3.8 years | 94.7% (Engel I) | 1/19 (5.3%) |
| **[9]** | **Cook (2004)** | University of California, Los Angeles (USA) | Anatomic hemispherectomy, functional hemispherectomy, lateral peri-insular hemispherotomy | 1986-2002 | 115 | 2 years | 71.2% ('seizure free') | 42/115 (36.5%) |
| **[10]** | **Delalande (2007)** | Fondation Ophtalmologique A. de Rothschild (France) | Vertical parasagittal hemispherotomy | 1990-2000 | 83 | 0.03-11.3 years | 74.1% (Engel I) | 13/83 (15.7%) |
| **[11]** | **Delvin (2003)** | Great Ormond Street Hospital (United Kingdom) | Modified functional and anatomic hemispherectomy | 1991-1997 | 33 | >1 year | 51.5% (Engel I) | 3/33 (9.1%) |
| **[12]** | **De Palma (2019)** | Niguarda Ca' Granda Hospital; Meyer Children Hospital; Bambino Gesù Children's Hospital (Italy) | Vertical parasagittal hemispherotomy, lateral peri-insular hemispherotomy | 2006-2016 | 92 | 0.1-16.6 years | 73.3% (Engel I) | 14/92 (15.2%) |
| **[13]** | **Dorfer (2013)** | Vienna Pediatric Epilepsy Center (Austria) | Vertical parasagittal hemispherotomy | 1998-2013 | 40 | >1 year | 91.9% (Engel I) | 1/40 (2.5%) |
| **[14]** | **Gaudio (2023)** | University Hospital Saint Luc (Belgium) | Vertical parasagittal sub-insular hemispherotomy | 2008-2023 | 25 | 0.3-14.9 years | 86.9% (Engel I) | 2/25 (8.7%) |
| **[15]** | **Iwasaki (2015)** | Tohoku University Graduate School of Medicine (Japan) | Lateral peri-insular hemispherotomy, vertical parasagittal hemispherotomy | 2001-2012 | 13 | >2 years | 84.6% (Engel I) | 0/13 (0%) |
| **[16]** | **Ji (2019)** | Peking University First Hospital (China) | Lateral peri-insular hemispherotomy | 2014-2017 | 83 | >2 years | 83.1% (Engel I) | NR |
| **[17]** | **Kalbhenn (2023)** | Evangelisches Klinikum Bethel (Germany) | Lateral peri-insular hemispherotomy, transsylvian lateral hemispherotomy | 2001-2018 | 140 | 2 years | 72.1% (Engel IA) | 17/140 (12.1%) |
| **[18]** | **Kumar (2015)** | Children’s Hospital Colorado (USA) | Lateral peri-insular hemispherotomy | 2002-2013 | 16 | 0.3-11.1 years | 81.3% (Engel I) | 4/16 (25.0%) |
| **[19]** | **Kwan (2010)** | Hospital for Sick Children (Toronto) | Hemidecordication, lateral peri-insular hemispherotomy | 1987-2007 | 41 | >2 years | 65.9% (Engel I/II) | 6/41 (14.6%) |
| **[20]** | **Lee (2013)** | Asan Medical Center (South Korea) | Functional hemispherectomy, anatomic hemispherectomy, lateral peri-insular hemispherotomy, vertical perisagittal hemispherotomy | 1997-2005 | 12 | 7.6-16.2 years | 66.7% (Engel I) | 5/12 (41.7%) |
| **[21]** | **Lew (2014)** | Medical College of Wisconsin (USA) | Functional hemispherectomy, anatomic hemispherectomy, lateral peri-insular hemispherotomy | 2004-2012 | 50 | 0.3-7.5 years | 80.0% (Engel I) | 11/43 (25.6%) |
| **[22]** | **Limbrick (2009)** | St. Louis Children’s Hospital (USA) | Functional hemispherectomy, lateral peri-insular hemispherotomy | 1995-2008 | 49 | 0.5-89 months | 77.6% (Engel I) | 5/49 (10.2%) |
| **[23]** | **Maehara (2002)** | Tokyo Metropolitan Neurological Hospital (Japan) | Lateral peri-insular hemispherotomy | 1994-1998 | 14 | >2 years | 42.9% (>90% seizure reduction) | 3/14 (21.4%) |
| **[24]** | **Marras (2010)** | Neurological Institute C. Besta (Italy) | Lateral peri-insular hemispherotomy | 2000-2007 | 13 | >2 years | 61.5% (Engel I) | 1/13 (7.7%) |
| **[25]** | **Moosa (2013)** | Cleveland Clinic (USA) | Anatomic, modified anatomic, and functional hemispherectomy | 1997-2009 | 170 | Mean: 5.3 years | 65.9% (Engel IA) | NR |
| **[26]** | **Nelles (2015)** | University of Bonn Medical Center (Germany) | Functional hemispherectomy | 2005-2013 | 34 | NR | 91.2% (Engel I) | NR |
| **[27]** | **Pacetti (2021)** | Istituto Giannina Gaslini; Niguarda Hospital (Italy) | Functional hemispherectomy, anatomic hemispherectomy, lateral peri-insular hemispherotomy | 2008-2018 | 14 | >1 year | 71.4% (Engel I) | 2/14 (14.3%) |
| **[28]** | **Panigrahi (2015)** | Krishna Institute of Medical Sciences (India) | Lateral peri-insular hemispherotomy, vertical parasagittal hemispherotomy | NR | 21 | >2 years | 90.5% (Engel I) | 1/21 (4.8%) |
| **[29]** | **Pinto (2014)** | Children’s Hospital Boston (USA) | Anatomic hemispherectomy, functional hemispherectomy, lateral peri-insular hemispherotomy | 1997-2011 | 36 | >1 year | 69.4% (Engel I) | 13/36 (36.1%) |
| **[30]** | **Ramantani (2013)** | University Hospital Freiburg (Germany) | Lateral peri-insular hemispherotomy, transsylvian lateral hemispherotomy | 2002-2011 | 52 | >1 year | 82.7% (Engel I) | 10/52 (19.2%) |
| **[31]** | **Ramantani (2023)** | University Children's Hospital Zurich (Switzerland); Hospital Fondation Adolphe de Rothschild (France); University Medical Center Utrecht (Netherlands); Great Ormond Street Hospital for Children (United Kingdom); University of Freiburg (Germany) | Lateral peri-insular hemispherotomy, vertical parasagittal hemispherotomy | 2000-2016 | 457 | >1 year | 75.3% (Engel IA) | 51/457 (11.2%) |
| **[32]** | **Rasmussen (1983)** | Montreal Neurologic Institute (Canada) | Functional hemispherectomy | 1952-1979 | 39 | >2 years | 43.6% (‘seizure free') | NR |
| **[33]** | **Schramm (2001)** | University of Bonn Medical Center (Germany) | Transsylvian hemispherotomy | NR | 16 | >1 year | 87.5% (Engel I) | 0/20 (0%) |
| **[34]** | **Schramm (2012)** | University of Bonn Medical Center (Germany) | Functional hemispherectomy, transsylvian hemispherotomy | 1990-2009 | 92 | >1 year | 84.8% (ILAE I) | 5/95 (5.2%) |
| **[35]** | **Shimizu (2000)** | Metropolitan Neurological Hospital (Japan) | Lateral peri-insular hemispherotomy | 1993-1999 | 27 | >1 year | 66.7% (Engel I) | 5/34 (14.7%) |
| **[36]** | **Shimizu (2005)** | Metropolitan Neurological Hospital (Japan) | Transopercular peri-insular hemispherotomy | 1983-2002 | 44 | >1 year | 65.9% (Engel I/II) | 5/44 (11.4%) |
| **[37]** | **Smith (1991)** | Montreal Neurologic Institute (Canada) | Functional hemispherectomy | 1976-1988 | 25 | >1 year | 72.0% (‘seizure free’) | NR |
| **[38]** | **Terra-Bustamante (2007)** | Ribeirão Preto Epilepsy Surgery Program (Brazil) | Functional hemispherectomy, lateral peri-insular hemispherotomy | 1996-2005 | 39 | >1 year | 53.8% (Engel I) | 1/39 (2.6%) |
| **[39]** | **Thomas (2012)** | Christian Medical College (India) | Lateral peri-insular hemispherotomy | 2000-2011 | 27 | Mean: 2.5 years | 92.6% (Engel I) | 1/27 (3.7%) |
| **[40]** | **van der Kolk (2013)** | Wilhelmina Children’s Hospital, University Medical Center Utrecht (Netherlands) | Functional hemispherectomy, lateral peri-insular hemispherotomy | 1996-2007 | 35 | 2 years | 85.7% (Engel I) | NR |
| **[41]** | **van Schooneveld (2016)** | University Medical Center Utrecht (Netherlands) | Hemispherectomy (not otherwise specified) | 1994-2009 | 31 | >5 years | 74.2% (Engel IA) | NR |
| **[42]** | **Verdinelli (2015)** | Swedish National Epilepsy Surgery Register (Sweden) | Functional hemispherectomy, lateral peri-insular hemispherotomy | 1995-2007 | 29 | 2 years | 55.2% (‘seizure free’) | NR |
| **[43]** | **Villarejo-Ortega (2013)** | Hospital Infantil Universitario Niño Jesús (Spain) | Functional hemispherectomy | 2001-2009 | 17 | >1 year | 58.8% (Engel I) | 1/17 (5.9%) |
| **[44]** | **Villemure (2006)** | Centre Hospitalier Universitaire Vaudois (Switzerland) | Lateral peri-insular hemispherotomy | NR | 37 | >1 year | 91.9% (Engel I) | 1/43 (2.3%) |
| **[45]** | **Weil (2020)** | Miami Children’s Hospital (USA) | Lateral peri-insular hemispherotomy | 2000-2014 | 69 | 2 years | 81.2% (Engel I) | 9/69 (13.0%) |
| **[46]** | **Yates (2023)** | Queensland Children’s Hospital (Australia) | Lateral peri-insular hemispherotomy | 2014-2020 | 13 | 0.5-3.8 years | 84.6% (Engel I) | 2/13 (15.4%) |

**Table S1.** Literature review of prior case series with emphasis on seizure outcomes and hydrocephalus.

**Supplemental References:**

^1.^ Abraham AP, Thomas MM, Mathew V, et al. EEG lateralization and seizure outcome following peri-insular hemispherotomy for pediatric hemispheric epilepsy. *Childs Nerv Syst*. 2019;35:1189-1195. doi:10.1007/s00381-019-04067-6

^2.^ Althausen A, Gleissner U, Hoppe C, et al. Long-term outcome of hemispheric surgery at different ages in 61 epilepsy patients. *J Neurol Neurosurg Psychiatry*. 2013;84(5):529-536. doi:10.1136/jnnp-2012-303811

^3.^ Arifin MT, Muttaqin Z, Hanaya R, et al. Hemispherotomy for drug-resistant epilepsy in an Indonesian population. *Epilepsy Behav Rep*. 2019;12:100337. doi:10.1016/j.ebr.2019.100337

^4.^ Basheer SN, Connolly MB, Lautzenhiser A, Sherman EMS, Hendson G, Steinbok P. Hemispheric Surgery in Children with Refractory Epilepsy: Seizure Outcome, Complications, and Adaptive Function. *Epilepsia*. 2007;48(1):133-140. doi:10.1111/j.1528-1167.2006.00909.x

^5.^ Buckley RT, Morgan T, Saneto RP, Barber J, Ellenbogen RG, Ojemann JG. Dysphagia after pediatric functional hemispherectomy. *J Neurosurg Pediatr*. 2014;13(1):95-100. doi:10.3171/2013.10.PEDS13182

^6.^ Caraballo R, Bartuluchi M, Cersósimo R, Soraru A, Pomata H. Hemispherectomy in pediatric patients with epilepsy: a study of 45 cases with special emphasis on epileptic syndromes. *Childs Nerv Syst*. 2011;27:2131-2136. doi:10.1007/s00381-011-1596-5

^7.^ Cats EA, Kho KH, van Nieuwenhuizen O, van Veelen CWM, Gosselaar PH, van Rijen PC. Seizure freedom after functional hemispherectomy and a possible role for the insular cortex: the Dutch experience. *J Neurosurg Pediatr*. 2007;107(4):275-280. doi:10.3171/PED-07/10/275

^8.^ Chandra P, Padma V, Shailesh G, Chandreshekar B, Sarkar C, Tripathi M. Hemispherotomy for intractable epilepsy. *Neurol India*. 2008;56(2):127-132. doi:10.4103/0028-3886.41988

^9.^ Cook SW, Nguyen ST, Hu B, et al. Cerebral hemispherectomy in pediatric patients with epilepsy: comparison of three techniques by pathological substrate in 115 patients. *J Neurosurg Pediatr*. 2004;100(2):125-141. doi:10.3171/ped.2004.100.2.0125

^10.^ Delalande O, Bulteau C, Dellatolas G, et al. Vertical parasagittal hemispherotomy. *Oper Neurosurg (Hagerstown)*. 2007;60(2):19-32. doi:10.1227/01.NEU.0000249246.48299.12

^11.^ Devlin AM. Clinical outcomes of hemispherectomy for epilepsy in childhood and adolescence. *Brain*. 2003;126(3):556-566. doi:10.1093/brain/awg052

^12.^ de Palma L, Pietrafusa N, Gozzo F, et al. Outcome after hemispherotomy in patients with intractable epilepsy: Comparison of techniques in the Italian experience. *Epilepsy Behav*. 2019;93:22-28. doi:10.1016/j.yebeh.2019.01.006

^13.^ Dorfer C, Czech T, Dressler A, et al. Vertical perithalamic hemispherotomy: A single‐center experience in 40 pediatric patients with epilepsy. *Epilepsia*. 2013;54(11):1905-1912. doi:10.1111/epi.12394

^14.^ del Gaudio N, Ferrao Santos S, Raftopoulos C. Modified Vertical Parasagittal Sub-Insular Hemispherotomy—Case Series and Technical Note. *Brain Sci*. 2023;13(10):1395. doi:10.3390/brainsci13101395

^15.^ Iwasaki M, Uematsu M, Osawa S ichiro, et al. Interhemispheric Vertical Hemispherotomy: A Single Center Experience. *Pediatr Neurosurg*. 2015;50(5):295-300. doi:10.1159/000437145

^16.^ Ji T, Liu M, Wang S, et al. Seizure Outcome and Its Prognostic Predictors After Hemispherotomy in Children With Refractory Epilepsy in a Chinese Pediatric Epileptic Center. *Front Neurol*. 2019;10. doi:10.3389/fneur.2019.00880

^17.^ Kalbhenn T, Cloppenborg T, Woermann FG, et al. Hemispherotomy in children: A retrospective analysis of 152 surgeries at a single center and predictors for long‐term seizure outcome. *Epilepsia*. 2023;64:1800-1811. doi:10.1111/epi.17632

^18.^ Kumar RM, Koh S, Knupp K, Handler MH, O’Neill BR. Surgery for infants with catastrophic epilepsy: an analysis of complications and efficacy. *Childs Nerv Syst*. 2015;31:1479-1491. doi:10.1007/s00381-015-2759-6

^19.^ Kwan A, Ng WH, Otsubo H, et al. Hemispherectomy for the Control of Intractable Epilepsy in Childhood: Comparison of 2 Surgical Techniques in a Single Institution. *Oper Neurosurg (Hagerstown)*. 2010;67:ons429-ons436. doi:10.1227/NEU.0b013e3181f743dc

^20.^ Lee YJ, Kim EH, Yum MS, Lee JK, Hong S, Ko TS. Long-Term Outcomes of Hemispheric Disconnection in Pediatric Patients with Intractable Epilepsy. *Journal of Clinical Neurology*. 2014;10(2):101-107. doi:10.3988/jcn.2014.10.2.101

^21.^ Lew SM, Matthews AE, Hartman AL, Haranhalli N. Posthemispherectomy hydrocephalus: Results of a comprehensive, multi-institutional review. *Epilepsia*. 2013;54(2):383-389. doi:10.1111/epi.12010

^22.^ Limbrick DD, Narayan P, Powers AK, et al. Hemispherotomy: efficacy and analysis of seizure recurrence. *J Neurosurg Pediatr*. 2009;4(4):323-332. doi:10.3171/2009.5.PEDS0942

^23.^ Maehara T, Shimizu H, Kawai K, et al. Postoperative development of children after hemispherotomy. *Brain and Development*. 2002;24(3):155-160. doi:10.1016/S0387-7604(02)00010-4

^24.^ Marras CE, Granata T, Franzini A, et al. Hemispherotomy and functional hemispherectomy: Indications and outcome. *Epilepsy Res*. 2010;89(1):104-112. doi:10.1016/j.eplepsyres.2009.09.006

^25.^ Moosa ANV, Gupta A, Jehi L, et al. Longitudinal seizure outcome and prognostic predictors after hemispherectomy in 170 children. *Neurology*. 2013;80(3):253-260. doi:10.1212/WNL.0b013e31827dead9

^26.^ Nelles M, Urbach H, Sassen R, et al. Functional hemispherectomy: postoperative motor state and correlation to preoperative DTI. *Neuroradiology*. 2015;57:1093-1102. doi:10.1007/s00234-015-1564-y

^27.^ Pacetti M, Giacomini T, Cossu M, et al. Hemispheric surgery for severe epilepsy in early childhood: a case series. *Epileptic Disord*. 2021;23(4):611-622. doi:10.1684/epd.2021.1303

^28.^ Panigrahi M, Krishnan SS, Vooturi S, Vadapalli R, Somayajula S, Jayalakshmi S. An observational study on outcome of hemispherotomy in children with refractory epilepsy. *Int. J. Surg*. 2016;36:477-482. doi:10.1016/j.ijsu.2015.05.049

^29.^ Pinto ALR, Lohani S, Bergin AMR, et al. Surgery for Intractable Epilepsy Due to Unilateral Brain Disease: A Retrospective Study Comparing Hemispherectomy Techniques. *Pediatr Neurol*. 2014;51(3):336-343. doi:10.1016/j.pediatrneurol.2014.05.018

^30.^ Ramantani G, Kadish NE, Brandt A, et al. Seizure control and developmental trajectories after hemispherotomy for refractory epilepsy in childhood and adolescence. *Epilepsia*. 2013;54(6):1046-1055. doi:10.1111/epi.12140

^31.^ Ramantani G, Bulteau C, Cserpan D, et al. Not surgical technique, but etiology, contralateral MRI, prior surgery, and side of surgery determine seizure outcome after pediatric hemispherotomy. *Epilepsia*. 2023;64(5):1214-1224. doi:10.1111/epi.17574

^32.^ Rasmussen T. Hemispherectomy for Seizures Revisited. *Can J Neurol Sci*. 1983;10(2):71-78. doi:10.1017/S0317167100044668

^33.^ Schramm J, Kral T, Clusmann H. Transsylvian Keyhole Functional Hemispherectomy. *Neurosurgery*. 2001;49(4):891-901. doi:10.1097/00006123-200110000-00021

^34.^ Schramm J, Kuczaty S, Sassen R, Elger CE, von Lehe M. Pediatric functional hemispherectomy: outcome in 92 patients. *Acta Neurochir (Wien)*. 2012;154(11):2017-2028. doi:10.1007/s00701-012-1481-3

^35.^ Shimizu H, Maehara T. Modification of Peri-insular Hemispherotomy and Surgical Results. *Neurosurgery*. 2000;47(2):367-373. doi:10.1097/00006123-200008000-00018

^36.^ Shimizu H. Our Experience with Pediatric Epilepsy Surgery Focusing on Corpus Callosotomy and Hemispherotomy. *Epilepsia*. 2005;46(s1):30-31. doi:10.1111/j.0013-9580.2005.461009.x

^37.^ Smith SJM, Andermann F, Villemure JG, Rasmussen TB, Quesney LF. Functional hemispherectomy. *Neurology*. 1991;41(11):1790-1794. doi:10.1212/WNL.41.11.1790

^38.^ Terra-Bustamante VC, Inuzuka LM, Fernandes RMF, et al. Outcome of hemispheric surgeries for refractory epilepsy in pediatric patients. *Childs Nerv Syst*. 2007;23:321-326. doi:10.1007/s00381-006-0212-6

^39.^ Thomas SG, Chacko AG, Thomas MM, Babu KS, Russell PSS, Daniel RT. Outcomes of Disconnective Surgery in Intractable Pediatric Hemispheric and Subhemispheric Epilepsy. *Int J Pediatr*. 2012;2012:527891. doi:10.1155/2012/527891

^40.^ van der Kolk NM, Boshuisen K, van Empelen R, et al. Etiology-specific differences in motor function after hemispherectomy. *Epilepsy Res*. 2013;103(2-3):221-230. doi:10.1016/j.eplepsyres.2012.08.007

^41.^ van Schooneveld MMJ, Braun KPJ, van Rijen PC, van Nieuwenhuizen O, Jennekens-Schinkel A. The spectrum of long-term cognitive and functional outcome after hemispherectomy in childhood. *Eur J Paediatr Neurol*. 2016;20(3):376-384. doi:10.1016/j.ejpn.2016.01.004

^42.^ Verdinelli C, Olsson I, Edelvik A, Hallböök T, Rydenhag B, Malmgren K. A long-term patient perspective after hemispherotomy – A population based study. *Seizure*. 2015;30:76-82. doi:10.1016/j.seizure.2015.05.016

^43.^ Villarejo-Ortega F, García-Fernández M, Fournier-Del Castillo C, et al. Seizure and developmental outcomes after hemispherectomy in children and adolescents with intractable epilepsy. *Childs Nerv Syst*. 2013;29:475-488. doi:10.1007/s00381-012-1949-8

^44.^ Villemure JG, Daniel RT. Peri-insular hemispherotomy in paediatric epilepsy. *Childs Nerv Syst*. 2006;22:967-981. doi:10.1007/s00381-006-0134-3

^45.^ Weil AG, Fallah A, Wang S, et al. Functional hemispherectomy: can preoperative imaging predict outcome? *J Neurosurg Pediatr*. 2020;25(6):567-573. doi:10.3171/2019.12.PEDS19370

^46.^ Yates CF, Malone S, Riney K, Shah U, Wood MJ. Peri-Insular Hemispherotomy: A Systematic Review and Institutional Experience. *Pediatr Neurosurg*. 2023;58(1):18-28. doi:10.1159/000529098
